# The effect of long-term unilateral deafness on the activation pattern in the auditory cortices of French-native speakers: influence of deafness side

**DOI:** 10.1186/1471-2202-10-23

**Published:** 2009-03-23

**Authors:** Julien Hanss, Evelyne Veuillet, Kamel Adjout, Julien Besle, Lionel Collet, Hung Thai-Van

**Affiliations:** 1 Université de Lyon, Lyon, F-69003, France; Université Lyon 1, Lyon, F-69003, France; 2 CNRS, UMR 5020, Neurosciences sensorielles-comportement-cognition, Lyon, F-69007, France; 3 Hospices Civils de Lyon, service d'audiologie et d'explorations orofaciales, hôpital Edouard Herriot, Lyon, F-69003, France; 4 Institut Fédératif des Neurosciences de Lyon, Lyon-Bron, F-69677, France; 5 INSERM U821, Dynamique cérébrale et cognition, Bron, F-69500, France

## Abstract

**Background:**

In normal-hearing subjects, monaural stimulation produces a normal pattern of asynchrony and asymmetry over the auditory cortices in favour of the contralateral temporal lobe. While late onset unilateral deafness has been reported to change this pattern, the exact influence of the side of deafness on central auditory plasticity still remains unclear. The present study aimed at assessing whether left-sided and right-sided deafness had differential effects on the characteristics of neurophysiological responses over auditory areas. Eighteen unilaterally deaf and 16 normal hearing right-handed subjects participated. All unilaterally deaf subjects had post-lingual deafness. Long latency auditory evoked potentials (late-AEPs) were elicited by two types of stimuli, non-speech (1 kHz tone-burst) and speech-sounds (voiceless syllable/pa/) delivered to the intact ear at 50 dB SL. The latencies and amplitudes of the early exogenous components (N100 and P150) were measured using temporal scalp electrodes.

**Results:**

Subjects with left-sided deafness showed major neurophysiological changes, in the form of a more symmetrical activation pattern over auditory areas in response to non-speech sound and even a significant reversal of the activation pattern in favour of the cortex ipsilateral to the stimulation in response to speech sound. This was observed not only for AEP amplitudes but also for AEP time course. In contrast, no significant changes were reported for late-AEP responses in subjects with right-sided deafness.

**Conclusion:**

The results show that cortical reorganization induced by unilateral deafness mainly occurs in subjects with left-sided deafness. This suggests that anatomical and functional plastic changes are more likely to occur in the right than in the left auditory cortex. The possible perceptual correlates of such neurophysiological changes are discussed.

## Background

Unilateral deafness represents a particular model for the investigation of functional auditory plasticity mechanisms in humans. In normal hearing subjects, the cortical activation pattern is characterized by shorter and larger neurophysiological responses over the hemisphere contralateral to the stimulated ear in response to monaural stimulation [1-3]. This activation pattern is thought to rely on a contralateral dominance in the auditory pathway: because the contralateral auditory pathway contains a greater number of nerve fibers than the ipsilateral, the former contributes to a more direct activation of the contralateral auditory cortex [4-6]. Cortical reorganization following profound unilateral deafness has been primarily reported in mammals. In cats, after cochlear ablation during the neonatal period, neurophysiological responses showed reduced activation thresholds in the auditory cortex ipsilateral to the intact ear [7]. Unilateral sound deprivation in adult mammals also leads to auditory pathway modifications: after unilateral hair cell destruction in adult guinea pigs, Popelar et al. [8] reported decreased activation thresholds and progressively increased amplitudes within two to three weeks in both the auditory cortex and inferior colliculus ipsilateral to the healthy ear.

In adult humans, previous data have revealed that auditory plasticity mechanisms also occur within the first weeks after the onset of unilateral deafness [9,10] and continue for several years [11]. In line with animal studies, the main changes occurring in the auditory cortex ipsilateral to the healthy ear of unilaterally deaf subjects have also been reported: using long latency auditory evoked potentials (late-AEPs), Ponton et al. [11] reported a more synchronous and more equal activation of both hemispheres resulting from an increased activation of the hemisphere ipsilateral to the healthy ear. Similarly, auditory evoked magnetic fields obtained with magnetoencephalography (MEG) showed stronger dipole moments in the hemisphere ipsilateral to the stimulated intact ear in subjects with late onset unilateral deafness [12] and congenital or early onset deafness [9]. While many studies have provided convincing evidence that cortical reorganization may be induced by unilateral deafness, only a very few of them have investigated the influence of the side of deafness on that reorganization. Recently, Khosla et al. [13] reported that, in response to monaural click stimulation, subjects with left-sided deafness (right ear stimulation) showed equal AEP amplitudes over the hemispheres, whereas subjects with right-sided deafness (left ear stimulation) showed normal amplitude asymmetry. The results of Khosla and colleagues, however, were obtained only with non-speech material. Further, they did not allow any conclusion about the potential influence of the side of deafness on inter-hemispheric AEP latencies' differences (or absence of differences). The results of a very recent study even suggest that unilateral deafness may not change AEP asymmetries [14]. Thus, the influence of the side of deafness on cortical reorganization still remains unclear. The present EEG study aimed at investigating whether left-sided and right-sided deafness had differential effects on the time course and amplitudes of auditory areas' responses to speech and non-speech stimuli. Eighteen unilaterally deaf subjects participated and were split into two groups depending on the side of deafness. All had a history of long-term profound unilateral deafness, with a mean duration of deafness above 5 years. Late-AEPs were elicited by non-speech and speech sounds delivered to the intact ear, then compared to data obtained in control subjects undergoing monaural stimulation. Inter-hemispheric differences in AEP amplitudes and latencies were analyzed, depending on the side of the deafness.

## Methods

This research was performed in compliance with the Helsinki Declaration and the protocol was approved by the local ethics committee ("Comité de protection des Personnes SUD-EST IV", reference number 03/009). Written consent was obtained from all participants.

### Subjects

**Figure 1:**
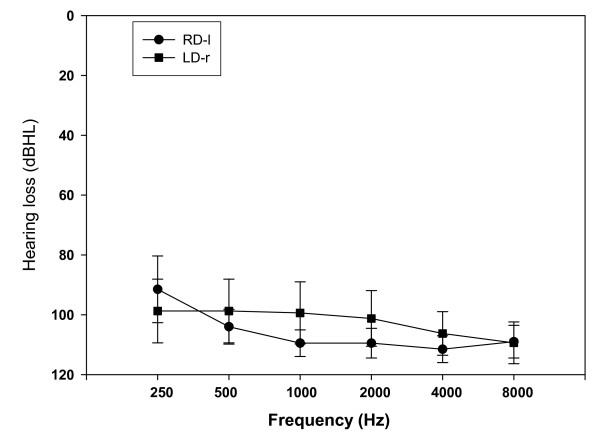
**Mean (± SEM) pure-tone bone conduction hearing thresholds (dB HL) obtained in the damaged ear of the eighteen unilaterally deaf subjects**. Profound deafness was found both in the LD-r and the RD-l group.

Thirty-four, (18 unilaterally deaf and 16 normal hearing) right-handed, French-native speakers aged from 27 to 59 years participated in the study. No subject reported any history of neurological impairment or language disorder. All patients had a profound unilateral deafness with bone conduction hearing thresholds on the deaf side worst than 65 dB HL for all frequencies tested between 250 and 8000 Hz (see Figure 1). Normal or subnormal air conduction hearing thresholds (< 25 dB HL) were obtained in their contralateral healthy ear. The control subjects had normal or subnormal air conduction hearing thresholds (< 25 dB HL) in both ears. All subjects were tested on one ear and split into four groups depending on the side of stimulation: normal-hearing subjects tested on the left ear (NH-l, n = 8; mean age ± standard error 42.62 ± 3.04 years; sex ratio= 4 M/4 F), subjects with right-sided deafness tested on the left ear (RD-l, n = 10; 40.3 ± 2.42 years; 8 M/2 F), normal hearing subjects tested on the right ear (NH-r, n = 8; mean age 39.37 ± 2.3 years; 5 M/3 F) and subjects with left-sided deafness tested on the right ear (LD-r, n = 8; 46.37 ± 3.7 years; 4 M/4 F). The groups did not differ significantly for mean age (ANOVA, p = 0.349). The groups of unilaterally deaf subjects did not differ for the mean duration of hearing loss (± standard error, SEM) at the time of testing, which was, respectively, 6.61 ± 3.7 years and 5.51 ± 3.3 years in the LD-r group and the RD-l group. Almost all hearing-impaired individuals (17 out of 18 patients) shared the same aetiology of deafness: i.e., sensorineural hearing loss. The deafness was indeed due to a sudden sensorineural hearing loss for 6 out of 8 patients in the LD-r group and for 8 out of 10 patients in the RD-l group. In addition, three other patients, two in the RD-l group and one in the LD-r group, had developed a sensorineural hearing loss due to an impairment of the inner ear consecutive to a chronic infection of the middle ear (cholesteatoma); in these three subjects, the inner ear damage was either due to the disease (spread of cholesteatoma towards the inner ear) or to the surgical technique. There was only one case of retrocochlear deafness in the LD-r group: this subject developed a sudden unilateral hearing loss after cochlear nerve resection during acoustic neuroma removal.

### Long latency auditory evoked potentials (late-AEPs)

**Figure 2:**
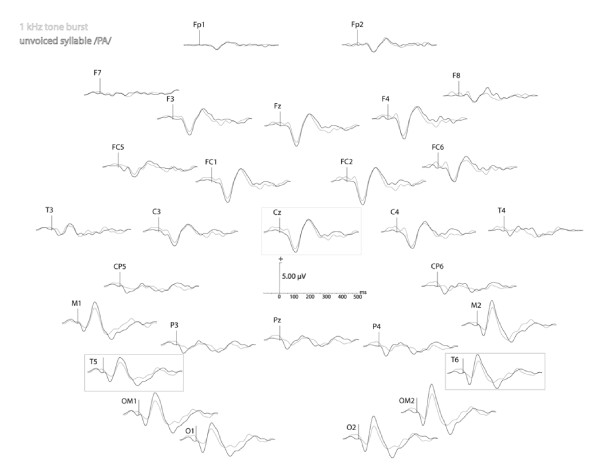
**Individual late-AEPs recorded from 29 scalp electrodes in response to 1 kHz tone burst and unvoiced syllable/pa/(data from one normal hearing subject stimulated on the left ear)**. The analysis is focused on the temporal electrodes (which exhibit a reversed waveform compared with Cz).

Scalp-EEG activity was recorded from 29 electrodes embedded in an electrode cap and placed in accordance with the international reference system (standard IFCN for digital recording of clinical EEGs: Fp1, Fp2, F7, F8, F3, Fz, F4, FC5, FC6, FC1, FC2, T3, C3, Cz, C4, T4, CP5, CP6, M1, M2, P3, Pz, P4, T5, T6, OM1, OM2, O1, O2 (see Figure 2)) using Micromed System Plus 98^® ^software. All electrodes were referenced to the tip of the nose and an electrode on the forehead served as the ground. Eye movements were monitored with a bipolar electrode montage. Impedance was maintained below 5 kΩ. Evoked potentials were recorded at a digitization rate of 1024 Hz as single epochs over an analysis window of 600 ms, which included 100 ms prior to presentation of the stimulus. Synchronous digital marking of the acoustic stimuli was obtained by connecting devices for EEG recording and auditory stimulation (Micromed trigger box).

Late-AEPs were elicited by two types of recorded sounds delivered monaurally to the subject's intact ear through earphones (TDH39): a non-speech sound -1 kHz tone-burst, total duration = 375 ms- and a speech-sound -voiceless consonant-vowel (CV) syllable/pa/, Voice Onset Time = +35 ms, total duration = 260 ms-. The stimulus intensity for EEG recordings was set at 50 dB SL (sensation level, relative to threshold at 1 kHz: that is, 50 dB above the hearing threshold at that frequency), delivered either to the healthy ear of deaf patients or the tested ear of control subjects. This intensity level was used to avoid any transcranial transfer effect to the contralateral ear in the control groups. The inter-stimulus interval (ISI) varied from 800 to 1200 ms. Six series of 100 stimuli (sequences of 50 1 kHz tone bursts, 50/pa/presented at random within each series) were repeated three times in a single session; as a result, each individual set of EEG data contained 1 800 responses (900 responses relative to each stimulation condition). Testing took place in a sound attenuated booth with the subjects seated in a comfortable reclining chair while watching a video tape (mute-subtitled mode). The neurophysiological recordings lasted for 90 minutes per subject.

### Data analysis

Each individual's AEP data were analyzed with ELAN^® ^software (O.Bertrand, INSERM U280, Lyon, France). Offline data were digitally band-pass filtered (1 to 30 Hz, 24 dB/octave slopes) and subjected to an automatic artefact rejection algorithm wherein sweeps containing activity exceeding ± 150 μV in any channel were excluded from subsequent analyses. Mean averaged waveforms for each eliciting stimulus were obtained separately for each subject. The neurophysiological approach focused on the early exogenous waves N100 and P150 of late-AEPs which are determined primarily by the physical properties of the stimuli contrary to the endogenous components associated with more cognitive processes [15]. Measurement of the exogenous peaks N100 (or N1) and P150 (or P2) focused on homologous temporal electrode pairs over the left (anterior: T3, posterior: T5) and right hemispheres (anterior: T4, posterior: T6). Inter-hemispheric latency differences (IHLD) were expressed as IHLD = (IL-CL), where CL and IL represent the latency values of the corresponding peaks in the hemisphere contralateral and ipsilateral, respectively, to the stimulated ear. Whereas positive IHLD values reflect shorter contralateral responses, an IHLD value tending towards zero reflects synchrony in the activation of the temporal lobes and negative values reflect shorter ipsilateral responses. Inter-hemispheric amplitude difference (IHAD) corresponded to (CA-IA)/(CA+IA), where CA and IA represent the amplitude values in the hemisphere contralateral and ipsilateral, respectively, to the stimulated ear. Whereas positive IHAD values reflect stronger contralateral responses, an IHAD value tending towards zero reflects symmetry over activation of the temporal lobes and negative values reflect stronger ipsilateral responses. The IHLD and IHAD of N1 and P2 components were compared between posterior temporal electrodes (T5 versus T6) where AEP show their maximum amplitude and where we expected the effects to be larger. Recordings from the anterior temporal electrodes T3 and T4 over patients and controls were less reproducible and are therefore presented as supplementary data.

### Statistical analysis

Analyses of variance (ANOVAs) were carried out to test the effect of group on the parameters measured (mean latency, amplitude and inter-hemispheric differences, i.e. IHLD and IHAD) for each stimulus. An additional post-hoc Bonferroni-adjusted t-test was performed on any significant ANOVAs. All mean values are presented with the standard error in brackets (+/- SEM).

## Results

### Long latency auditory evoked potentials via the posterior temporal electrodes T5-T6

**Figure 3:**
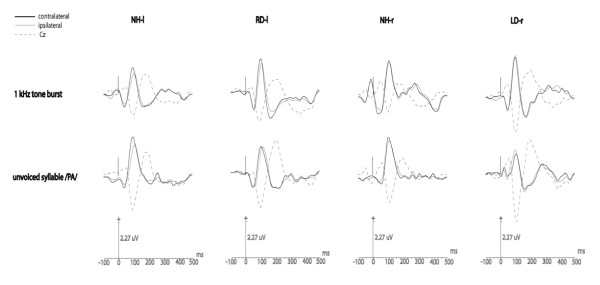
**Grand average of individual late-AEP responses relative to each stimulus over the temporal lobes ipsilateral and contralateral to the stimulation (corresponding grand average at Cz is shown as a dotted line: the waveform is reversed compared with that at the temporal lobes and serves the as reference)**.

Figure 3 shows the grand averaged waveforms of late-AEP responses for each group of subjects relative to each stimulus over the temporal lobes ipsilateral and contralateral to the stimulation with averaged waveforms recorded from the electrode site Cz (central electrode which serves as the reference for the detection of AEP peaks).

**Figure 4:**
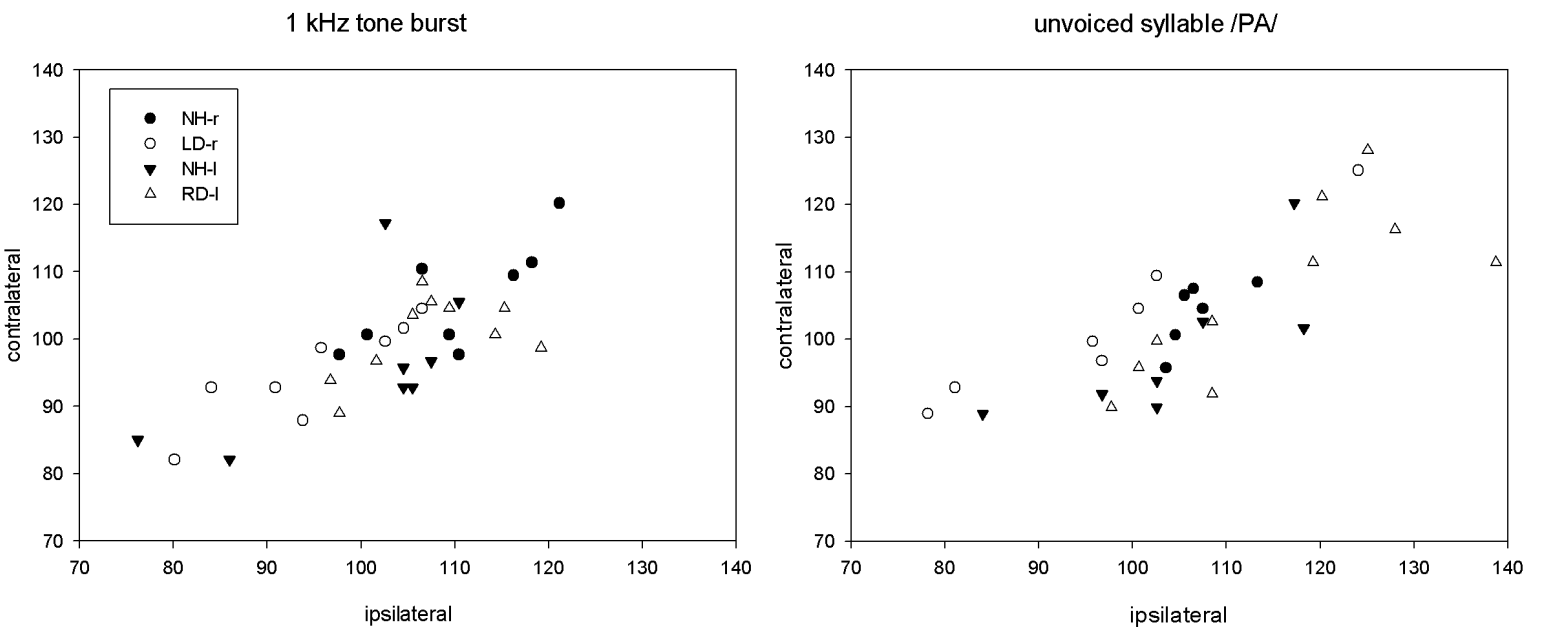
**Individual N1 latency for each stimulus over the temporal lobes ispilateral and contralateral to the stimulation**.

**Figure 5:**
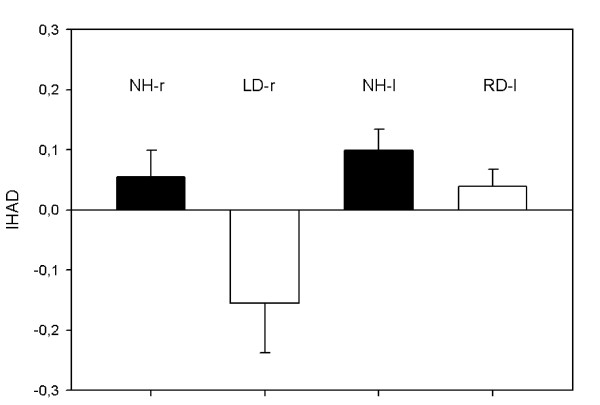
**Inter-hemispheric amplitude difference for N1-P2 complex over the posterior temporal lobes (electrodes T5 and T6) in response to the unvoiced syllable/pa/**. The LD-r group shows a significant reversed asymmetry in favour of the ipsilateral cortex (healthy-side dominance) compared with NH-r controls.

In order to simplify presentation of the results, data are shown separately for each group of subjects. Figure 4 and Table 1 show, respectively, the individual and mean values of N1 latency over the temporal lobes ipsilateral and contralateral to the stimulation and the corresponding N1 IHLD mean values, for each stimulus. Table 2 shows N1-P2 complex amplitude mean values over the temporal lobes ipsilateral and contralateral to the stimulation and corresponding IHAD mean values, relative to each stimulus. Figure 5 shows IHAD mean values in response to the unvoiced syllable/pa/.

**Table 1:** Mean N1 latency and N1 IHLD (± SEM) over posterior temporal lobes (electrodes T5 and T6) for each stimulation (the number of subjects within each group is in brackets).

	**NH-l**	**RD-l**	**NH-r**	**LD-r**	**ANOVA**
**1 kHz tone burst**					
Ipsilateral	99.654 ± 4.235	107.372 ± 2.355	110.035 ± 2.936	94.769 ± 3.384	F(3.30) = 4.632, p = 0.009* *
	(8)	(10)	(8)	(8)	
Contralateral	95.990 ± 3.959	100.533 ± 1.907	106.005 ± 2.854	95.013 ± 2.661	F(3.30) = 2.958, p = 0.048*
	(8)	(10)	(8)	(8)	
IHLD	3.664 ± 3.574	6.839 ± 2.111	4.030 ± 1.975	-0.244 ± 1.628	*F(3.30) = 1.508, p = 0.232*
	(8)	(10)	(8)	(8)	

**Unvoiced CV syllable/pa/**					
Ipsilateral	104.120 ± 4.487	114.895 ± 4.247	106.819 ± 1.420	97.002 ± 5.747	F(3.26) = 3.046, p = 0.047*
	(7)	(10)	(6)	(7)	
Contralateral	98.398 ± 4.165	106.786 ± 4.065	103.888 ± 1.981	102.445 ± 4.580	*F(3.26) = 0.800, p = 0.505*
	(7)	(10)	(6)	(7)	
IHLD	5.722 ± 2.950	8.109 ± 2.794	2.931 ± 1.405	-5.443 ± 1.717	F(3,26) = 5.599, p = 0.004* *
	(7)	(10)	(6)	(7)	

**Table 2:** N1-P2 complex amplitude mean values and corresponding IHAD (± SEM) over temporal lobes (electrodes T5 and T6) for each stimulation (the number of subjects within each group is in brackets).

	**NH-l**	**RD-l**	**NH-r**	**LD-r**	**ANOVA**
**1 kHz tone burst**					
Ipsilateral	2.724 ± 0.513	3.318 ± 0.434	3.931 ± 1.155	4.337 ± 0.776	*F(3.26) = 1.077, p = 0.376*
	(7)	(10)	(5)	(8)	
Contralateral	3.486 ± 0.519	3.973 ± 0.483	4.830 ± 1.212	4.165 ± 0.668	*F(3,25) = 0.535, p = 0.663*
	(7)	(10)	(4)	(8)	
IHAD	16.7 ± 7.05	9.20 ± 2.77	0.851 ± 8.39	1.06 ± 6.57	*F(3.25) = 1.563, p = 0.223*
	(7)	(10)	(4)	(8)	

**Unvoiced CV syllable/pa/**					
Ipsilateral	2.533 ± 0.401	3.114 ± 0.479	2.783 ± 0.807	3.229 ± 0.501	*F(3.25) = 0.354, p = 0.786*
	(7)	(10)	(5)	(7)	
Contralateral	3.097 ± 0.512	3.225 ± 0.416	3.363 ± 1.135	2.666 ± 0.722	*F(3.25) = 0.204, p = 0.893*
	(7)	(10)	(5)	(7)	
IHAD	9.90 ± 3.49	3.95 ± 2.77	5.51 ± 4.45	-15.5 ± 8.24	F(3.25) = 4.956, p = 0.008* *
	(7)	(10)	(5)	(7)	

The comparison between ipsi and contralateral responses for both groups of normal hearing subjects (NH-r and NH-l) revealed shorter contralateral N1 mean latency with positive IHLD (Table 1) and larger contralateral N1-P2 complex amplitudes with the corresponding positive IHAD (Table 2). These results reflected, respectively, an earlier and a stronger activation over the contralateral cortex, for both types of stimulation.

Whatever the stimulation condition, there was no difference between the RD-l group and normal hearing subjects for all measured parameters: the RD-l group exhibited shorter N1 mean latency over the contralateral cortex with positive IHLD mean values (even larger when compared with controls but not statistically significant), i.e. the normal pattern of asynchrony over the temporal lobes. Moreover, the RD-l group showed larger mean amplitudes of N1-P2 complexes over the contralateral cortex with positive IHAD mean values (reduced but preserved compared to the controls (non significant)), reflecting a normal pattern of asymmetry over the temporal lobes.

In contrast, important neurophysiological differences were observed in the LD-r group compared to the three other groups. In response to non-speech sound (1 kHz tone-burst), the mean value of N1 IHLD which tended towards 0 was lower than the values obtained in the other groups (Table 1) suggesting that in this group, the activation over the right and left auditory areas was more synchronous. This said, the one-way ANOVA did not show any significant difference between groups (F_3.30 _= 1.508; p = 0.232). In spite of a significant group effect for contralateral N1 latency (F_3.30 _= 2.958; p = 0.048), the post-hoc multiple comparison procedure also did not show any significant differences between groups. However, the one-way ANOVA revealed a main group effect on the N1 latency ipsilateral to the stimulated ear (F_3.30 _= 4.632; p = 0.009). The post-hoc multiple comparison test revealed a significantly shorter N1 ipsilateral latency in the LD-r group compared to both the NH-r (t_15.266 _= 3.262, p = 0.017) and RD-l (t_12.603 _= 2.839, p = 0.048) groups. With amplitude, the ANOVAs did not show any group effect either for N1-P2 mean amplitude values or the corresponding IHAD mean values (Table 2). The results however, showed a trend towards larger amplitudes over the ipsilateral cortex. Furthermore, corresponding IHAD mean values tended towards zero, i.e. activation over the left and right temporal lobes was more symmetrical.

In response to speech-sound (unvoiced syllable/pa/), neurophysiological changes exhibited by the LD-r group were more pronounced. The ANOVA revealed a significant group effect on N1 IHLD (F_3.26 _= 5.599, p = 0.004): the N1 IHLD mean value of the LD-r group was negative, significantly reversed compared to both RD-l (t_13.552 _= 3.964, p = 0.003) and the controls NH-l (t_11.166 _= 3.011, p = 0.034). Furthermore, whereas the groups did not significantly differ for contralateral N1 latency, a one way ANOVA revealed a significant group effect on ipsilateral N1 latency (F_3.26 _= 3.046, p = 0.047). Multiple comparisons revealed a significantly shorter ipsilateral N1 latency in the LD-r group compared to the RD-l group (t_17.893 _= 2.958, p = 0.039). In response to speech-sound, the ANOVA revealed a group effect on the N1-P2 complex IHAD mean value (F_3.25 _= 4.956, p = 0.008): the LD-r group showed a negative IHAD mean value, significantly reversed compared to the NH-l group (t_0.254 _= 3.560, p = 0.009). With amplitude mean values, the healthy-side dominance of the N1-P2 complex exhibited by the LD-r group seemed to result from a combined contralateral decrease/ipsilateral increase in activity compared with controls stimulated in the same ear (NH-r) (non significant).

As shown in Table 3, supplementary data from the homologous anterior temporal electrodes T3 and T4 showed similar neurophysiological profiles among groups. A one factor ANOVA revealed for T3-T4 a group effect on N1 IHLD in response to both non-speech and speech sounds. The LD-r group exhibited a significant reversal asynchrony in favour of the ipsilateral cortex compared with the RD-l group (p = 0,004) in response to tone burst and compared with both the NH-r (p = 0,006) and RD-l (p = 0,008) groups' in response to the unvoiced syllable/pa/. These results reinforce the neurophysiological changes observed in the posterior temporal electrodes in the LD-r group.

**Table 3:** Mean N1 latency and N1 IHLD (± SEM) over anterior temporal lobes (electrodes T3 and T4) for each condition of stimulation (the number of subjects within each group is in brackets).

	**NH-l**	**RD-l**	**NH-r**	**LD-r**	**ANOVA**
**1 kHz tone burst**					
Ipsilateral	102.911 ± 5.081	111.378 ± 2.467	110.279 ± 3.361	95.380 ± 3.973	F(3.28) = 4.563, p = 0.010*
	(6)	(10)	(8)	(8)	
Contralateral	97.212 ± 5.639	101.981 ± 2.557	104.051 ± 2.442	97.578 ± 3.817	*F(3.28) = 0.887, p = 0.460*
	(6)	(10)	(8)	(8)	
IHLD	5.667 ± 4.918	9.397 ± 1.840	6.228 ± 1.405	-2.198 ± 1.943	F(3.27) = 5.042, p = 0.007* *
	(5)	(10)	(8)	(8)	

**Unvoiced CV syllable/pa/**					
Ipsilateral	104.539 ± 2,585	105.795 ± 4.689	103.725 ± 4.110	96.025 ± 6.734	*F(3,20) = 0.768, p = 0.526*
	(4)	(7)	(6)	(7)	
Contralateral	91.840 ± 2.610	96.444 ± 3.947	102.096 ± 3.832	107.470 ± 5.669	*F(3,21) = 2.593, p = 0.080*
	(6)	(7)	(6)	(6)	
IHLD	13.187 ± 4.307	9.351 ± 2.539	0.586 ± 4.398	-8.956 ± 3.984	F(3.18) = 7.008, p = 0.003* *
	(4)	(7)	(5)	(6)	

## Discussion

Cortical reorganization following unilateral deafness has been reported using different techniques in adult humans. However, the influence of the side of deafness on auditory cortical plasticity remains unclear. This study provides evidence that left and right-sided deafness have differential effects on neurophysiological responses at the cortical level.

In both control groups (NH-r and NH-l), monaural stimulation with non-speech and speech sounds produced a normal pattern of asynchrony and asymmetry over the temporal lobes in favour of the contralateral cortex. This result agrees with data in the literature regarding the cortical activation pattern of normal hearing subjects in response to monaural stimulation [1-3]. Late-AEP responses evoked by a 1 kHz tone burst, however, showed with the NH-l group a large but non significant asymmetry in favour of the contralateral cortex compared with the NH-r group. Such differences between right and left ear stimulation have been previously reported with pure tones in normal hearing subjects. Recent AEP data (auditory N100) from Hine et al. [14] revealed, in normal hearing subjects stimulated with 1 kHz tone and white noise, a more contralaterally dominant activity for left compared to right ear stimulation. Whereas monaural stimulation with 1 kHz tones yielded a significantly stronger mean N100m dipole moment over the contralateral hemisphere in response to left-ear stimulation, the mean N100m dipole moment was stronger over the ispilateral hemisphere for right-ear stimulation (MEG data from Vasama and Makela [12]). Likewise, with monaural stimulation using a 1 kHz sine tone, the lateralization ratio (contralateral/ispilateral) of BOLD signals was larger when the left ear was stimulated than the right [16]. These results may reflect the higher specialization of the right hemisphere -more direct activation pathways with left ear stimulation – for processing tones and music [17-19].

In unilaterally deaf subjects, the modifications of the activation timing over auditory areas depended on the side of deafness. In the RD-l group, stimulation of the healthy ear with non-speech and speech sounds produced a normal pattern of asynchrony over the auditory areas; asynchrony in favour of the contralateral cortex was even more pronounced (non significant) in both conditions compared with the NH-l control group. In contrast, stimulation of the healthy ear in the LD-r group produced an abnormal activation pattern characterized by synchrony over the auditory areas in response to non-speech sound and even a significant reversal pattern in favour of the ipsilateral cortex in response to speech sound by a significant shortening of the N1 ipsilateral latency. Although this was not the key result of their studies, the MEG data from Vasama and Makela [12] and Fujiki et al. [9] also revealed differential effects of the side of unilateral deafness on neurophysiological changes over the auditory areas. Stimulating the healthy ear with 1 kHz tone, Vasama and Makela [12] reported significantly shorter ipsilateral N100m mean latency in subjects with left-sided deafness; in contrast, N100 mean latency was shorter over the contralateral hemisphere in subjects with right-sided deafness. Likewise, when Fujiki et al. [9] stimulated the healthy ear with 1 kHz tone bursts and the vowel/a/, only subjects with left-sided deafness showed a significantly reduced N100m IHLD compared with controls. Using AEP (N100) recordings in response to 1 kHz tone and white noise, Hine et al. [14] observed in 3 out of 4 patients with left-sided deafness (right ear stimulation) less inter-hemispheric latency differences compared to patients with right-sided deafness (left ear stimulation). Nevertheless, these authors did not carry out group analyses.

Although Khosla et al., using click stimuli delivered at an intensity level close to the one we used (70 dB HL), did not report any influence of the deafness side on late-AEP latencies, they showed clear differential ear effects when it came to late AEP amplitudes. Stimulating the healthy ear, they reported that inter-hemispheric amplitude differences of Root Mean Square, N1b-P2 and Ta-Tb complexes were the same in both controls and subjects with right-sided deafness; in contrast, subjects with left-sided deafness showed significantly reduced inter-hemispheric differences for all these parameters, i.e. a more symmetrical activation pattern over hemispheres [13]. In our study, modifications in the degree of activation over auditory areas also depended on the side of the deafness. In the RD-l group, in response to non-speech and speech sounds, the amplitude asymmetry in favour of the auditory cortex contralateral to the stimulated ear was preserved. In contrast, stimulation of the healthy ear in subjects with left-sided deafness produced an abnormal activation pattern characterized by amplitude symmetry in response to non speech sound (non significant) and even a significant reversal pattern in favour of the ipsilateral cortex in response to speech sound.

Overall, our profiles of neurophysiological responses elicited in subjects with RD did not differ from those of normal hearing subjects: a "normal" activation pattern over auditory areas may suggest a weak degree of cortical reorganization following RD. Conversely, important modifications of both timing and degree of activation in auditory areas were reported in subjects with LD: the more symmetrical activation pattern in response to pure tone and especially the reversal pattern in favour of the ispilateral hemisphere in response to speech sound may reflect a high potency of cortical reorganization following LD. These results are in agreement with those reported by Khosla et al. [13] who suggested that auditory cortical plasticity mainly occurred in left-sided deafness. Moreover, consistent with previous data obtained both in animals [7,8] and humans [9-13,20], our neurophysiological results suggest that plasticity mechanisms mainly involve the auditory cortex ipsilateral to the healthy ear. Such cortical changes are likely to be related to the modifications observed at sub-cortical levels in animals. After unilateral cochlear ablation in adult gerbils, synaptic inhibition was predominantly decreased within the inferior colliculus ipsilateral to the healthy ear [21,22]. In the same line, measuring the expression of the growth-associated GAP43 protein after unilateral deafening in adult rats, Illing et al. [23] reported an increased excitatory synaptogenesis within the ventral cochlear nucleus (VCN) on the affected side, i.e. the brainstem structure that projects mainly into the inferior colliculus ipsilateral to the healthy-side. In contrast, neurons from the affected-side of the VCN projected inhibitory pathways on the opposite cochlear nucleus. Thus, there would be an active process of reorganization to compensate for the loss of sensory inputs on the deaf side; it may stem from bottom-up and top-down mechanisms. Interestingly, the hemispheric differences in sound processing have been related to cytoarchitectural differences between the temporal lobes, the left temporal lobe showing more myelinated axons [24], with larger pyramidal cells, wider columns and denser afferent innervation [25]. Even speculative, a possible interpretation of our neurophysiological results (showing auditory cortical reorganization in the right hemisphere of subjects with left-sided deafness) is that anatomical and functional plastic changes are more likely to occur in the right than in the left auditory cortex, the right temporal lobe exhibiting a higher potency of re-myelinization and afferentation.

### What could be the perceptual correlates of such modifications of the cortical activation pattern over auditory areas?

Whereas unilateral deafness is well known to significantly alter the perceptual performances of the intact ear in spatial sound localization [26-29] and sound recognition with competing background noise [29-32], little is known about the consequences of unilateral deafness on basic sound processing. Using gap detection task, Sininger and de Bode [33] did not report differences on the gap detection thresholds between the intact ear of unilaterally deaf subjects and the corresponding ear of normal hearing subjects depending on the side of stimulation. However, the absence of asymmetry over the temporal lobes has been previously associated with impairment in the perception of acoustic cues in normal hearing subjects. Combining neurophysiological (late-AEPs) and psychoacoustical approaches in normal hearing English-native speakers, Bellis et al. [34] reported that elderly subjects, when compared to children and young adults, demonstrated less asymmetry (amplitude of P1N1 complex) in the temporal lobes and that this was associated with a reduced ability to discriminate speech syllables differing in the onset frequency of the third formant (F3). The authors consequently suggested that with monaural stimulation, the absence of asymmetry over the temporal lobes may affect the perception of acoustic cues involving fine spectrotemporal resolution and may underlie, at least partially, the speech perception difficulties presented by ageing adults. In the same line, Eichele et al. [35] showed that in normal hearing subjects, the asymmetry of N100 latency over temporal lobes -i.e. N100 asynchrony- predicted the ear advantage in dichotic listening. Even speculative, it is reasonable to assume that the loss of asynchrony/asymmetry observed in subjects with left-sided deafness may also have consequences on the perception of acoustic features by the intact ear. Hence, further research using neurophysiological recordings during active behavioural tasks is needed to investigate perceptual correlates of auditory cortical plasticity in unilaterally deaf subjects.

## Conclusion

Numerous studies have reported cortical reorganization following unilateral deafness in both mammals and humans. However, the influence of the deafness side on auditory cortical plasticity remains unclear in humans. The results of the study show that cortical reorganization induced by unilateral deafness mainly occurs in subjects with left-sided deafness, consistent with previous data from Khosla et al. [13]. Moreover, it suggests that anatomical and functional plastic changes are more likely to occur in the right than in the left auditory cortex. The combination of speech evoked responses at both the brainstem (speech auditory brainstem responses) and cortical levels would certainly advance our knowledge of auditory plasticity mechanisms in unilaterally deaf subjects. Moreover, further investigation is needed to assess to what extent both asynchrony and asymmetry over the auditory areas could represent electrophysiological correlates of speech perception in unilaterally deaf and even normal hearing subjects.

## Authors' contributions

LC, HTV and EV conceived of the study, participated in its design and coordination and have been involved in drafting the manuscript. KA made substantial contributions to acquisition and analysis of data. JB helped substantially in the analysis and interpretation of data under ELAN software. JH, first author, was involved in acquisition, analysis and interpretation of data and wrote the manuscript
